# A Novel Multi-Exon Deletion of *PACS1* in a Three-Generation Pedigree: Supplements to *PACS1* Neurodevelopmental Disorder Spectrum

**DOI:** 10.3389/fgene.2021.690216

**Published:** 2021-07-23

**Authors:** Yuan Liu, Hongke Ding, Tizhen Yan, Ling Liu, Lihua Yu, Yanlin Huang, Fake Li, Yukun Zeng, Weiwei Huang, Yan Zhang, Aihua Yin

**Affiliations:** ^1^Medical Genetics Centre, Guangdong Women and Children Hospital, Guangzhou, China; ^2^Prenatal Diagnosis Centre, Guangdong Women and Children Hospital, Guangzhou, China; ^3^Department of Medical Genetics, Liuzhou Key Laboratory of Birth Defects Prevention and Control, Liuzhou Maternity and Child Healthcare Hospital, Liuzhou, China

**Keywords:** Schuurs-Hoeijmakers syndrome, *PACS1*, multi-exon deletion, loss of function, rare disease, *PACS1* neurodevelopmental disorder

## Abstract

*PACS1* neurodevelopmental disorder (*PACS1*-NDD) is a category of rare disorder characterized by intellectual disability, speech delay, dysmorphic facial features, and developmental delay. Other various physical abnormalities of *PACS1*-NDD might involve all organs and systems. Notably, there were only two unique missense mutations [c.607C > T (p.Arg203Trp) and c.608G > A (p.Arg203Gln)] in *PACS1* that had been identified as pathogenic variants for *PACS1*-NDD or Schuurs-Hoeijmakers syndrome (SHMS). Previous reports suggested that these common missense variants were likely to act through dominant-negative or gain-of-function effects manner. It is still uncertain whether the intragenic deletion or duplication in *PACS1* will be disease-causing. By using whole-exome sequencing, we first identified a novel heterozygous multi-exon deletion covering exons 12–24 in *PACS1* (NM_018026) in four individuals (two brothers and their father and grandfather) in a three-generation family. The younger brother was referred to our center prenatally and was evaluated before and after the birth. Unlike SHMS, no typical dysmorphic facial features, intellectual problems, and structural brain anomalies were observed among these four individuals. The brothers showed a mild hypermyotonia of their extremities at the age of 3 months old and recovered over time. Mild speech and cognitive delay were also noticed in the two brothers at the age of 13 and 27 months old, respectively. However, their father and grandfather showed normal language and cognitive competence. This study might supplement the spectrum of *PACS1*-NDD and demonstrates that the loss of function variation in *PACS1* displays no contributions to the typical SHMS which is caused by the recurrent c.607C > T (p.Arg203Trp) variant.

## Introduction

*PACS1* neurodevelopmental disorder (*PACS1*-NDD) or Schuurs-Hoeijmakers syndrome (SHMS; MIM#615009) is a rare disease with typical mild-to-severe neurodevelopmental delays and mental retardation. Most of the reported patients were found to have three distinct types of traits: dysmorphic facial features, developmental delay (DD), and a range of mild-to-severe intellectual disability (ID). Other common clinical features include feeding difficulty, hypotonia, epilepsy, autism, congenital heart diseases, and ocular anomalies (Lusk et al., 2020; Tenorio-Castano et al., 2021).

As far as we know, SHMS is an autosomal dominant disorder which is frequently caused by a recurrent variation in phosphofurin acidic cluster sorting protein 1 (*PACS1*) gene on chromosome 11q13 region (Schuurs-Hoeijmakers et al., 2012; Lusk et al., 2020). *PACS1* (NM_018026.3) encompassing a total of 24 exons, and the pathogenic variation for SHMS locates in exon 4 which was initially described and identified in 2012 (Schuurs-Hoeijmakers et al., 2012). To date, only two single nucleotide variations in *PACS1* have been reported as the cause of SHMS. A heterozygous *de novo* variation c. 607C > T (p.Arg203Trp) in *PACS1* is present in almost all of the reported cases (Kurt Colak et al., 2020; Lusk et al., 2020; Seto et al., 2020), except another variation c.608G > A (p.Arg203Gln) which affects the same amino acid (Miyake et al., 2018). Since Schuurs-Hoeijmakers first characterized the recurrent *de novo* variation of *PACS1* (c.607C > T) in two unrelated individuals in 2012 (Schuurs-Hoeijmakers et al., 2012), approximately 63 cases with SHMS have been described in the literature until now, including 60 postnatal individuals and three prenatal cases (Kurt Colak et al., 2020; Lusk et al., 2020; Seto et al., 2020; Gana et al., 2021; Tenorio-Castano et al., 2021).

As reported previously, this common missense variant was likely to act through gain-of-function effects manner or dominant-negative mutational mechanisms which could explain the typical facial similarity in those patients (Schuurs-Hoeijmakers et al., 2012, 2016; Miyake et al., 2018). Definite evidence of this hypothesis needs further functional studies. Moreover, the effects of other types of variants in *PACS1* still remain unclear due to the limited evidence regarding the relevance between the intragenic deletion/duplication of *PACS1* and *PACS1*-NDD. Here, by using whole-exome sequencing (WES), we first report two brothers and two adults in a three-generation family with a novel heterozygous multi-exon deletion variation in *PACS*. They showed mild hypermyotonia of their extremities at 3 months of age and recovered over time. Only the brothers showed mild speech and cognitive delay, and no other SHMS-related phenotypes were found among these four cases. Our study demonstrated that this novel multi-exon deletion of *PACS1* might display no contribution to SHMS.

## Case Presentation

A 29-year-old Chinese woman (G3P1A1L1) was referred to our center for prenatal counseling at over 12 weeks of gestation for increased nuchal translucency (NT; 3.4 mm) in the current fetus. It was the third pregnancy for this non-consanguineous couple, who previously had one normal delivery and one abortion due to increased NT and cervical lymphatic hygroma at 14 weeks of pregnancy. She got pregnant naturally for the current pregnancy and reported no other remarkable medical history. Maternal serum screening for Down syndrome and TORCH (*Toxoplasma gondii*, *Rubella* virus, *cytomegalovirus*, and *Herpes simplex* virus) infection showed low-risk results. After genetic counseling, the couple declined non-invasive prenatal testing and finally decided to receive an invasive procedure of chorionic villus sampling at more than 12 weeks of gestation. Chromosomal microarray analysis (CMA) showed that the fetus had no chromosomal micro-duplication or deletion. By using WES, we identified a novel heterozygous multi-exon deletion covering exons 12–24 in *PACS1* (NM_018026) in the fetus.

This case was presented in a multidisciplinary team discussion with clinical geneticist, perinatologist, and neonatologist. An optimal approach including genetic counseling, prenatal diagnostic procedure, and risk assessment for both the mother and fetus was made. After much discussion, the couple decided to keep the pregnancy. No structural anomalies were observed by ultrasound examination at the 24th and 28th week of her pregnancy, respectively. The proband (current fetus) was born at 40 weeks of gestation age, and the Apgar score was 9 at 1 and 5 min. However, on day 5 after the delivery, the proband was referred to be hospitalized due to hyperbilirubinemia (the serum total bilirubin value was 262.5 μmol/L). A cranial ultrasound revealed an image suggestive of left choroid plexus hemorrhage and increased middle cerebral artery resistance index (RI) and pulsatility index (PI). No other abnormalities were encountered. At the age of 3 months, mild hypermyotonia of his extremities was noted when the proband received routine newborn assessment. A magnetic resonance imaging (MRI) brain scan showed no intraventricular hemorrhage and structural brain abnormalities. Then, he was treated with citicoline and mecobalamin, and the neurodevelopmental assessment at the age of 6 months was normal. The elder brother also showed mild hypermyotonia of his extremities also at 3 months of age and recovered over time. At the time of this submission, the age of the proband and his brother was 13 and 27 months old, respectively. According to the latest neurodevelopmental assessment, mild speech and cognitive delay were noticed in the two brothers. The brothers also received other assessments regarding different organs; no remarkable abnormality was encountered. The proband’s grandfather was a retired worker, and the proband’s father was 32 years old and graduated from a university with a bachelor’s degree in architecture. No congenital malformations, ID, and neurological abnormalities were noted from the proband’s grandfather and father.

## Laboratory Investigations and Results

### CMA Analysis

**FIGURE 1:**
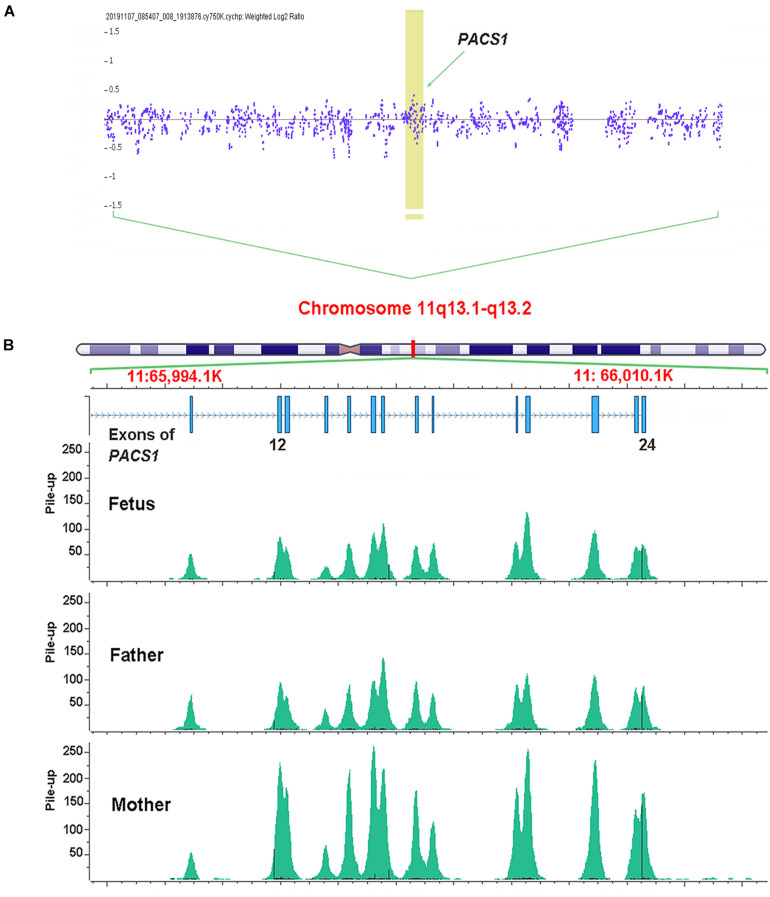
Genomic DNA extracted from chorionic villus tissue was used for single-nucleotide polymorphism array analysis. No micro-duplication or deletion was detected on the region of chromosome 11q3.1–11q3.2. The arrow indicates the sites of *PACS1* **(A)**. Whole-exome sequencing identified a multi-exon deletion (EX12_24del) in *PACS1* which was located on chromosome 11q13. The picture of pile-up reads overlapping the sites was generated by Golden Helix Genome Browse. The fetus and the father showed about half pile-up reads to that of the mother that indicated a heterozygous deletion among this region **(B)**.

Genomic DNA extracted from chorionic villus tissue and the peripheral blood of parents was used for routine prenatal genetic testing. A single-nucleotide polymorphism CMA analysis was performed based on the Affymetrix CytoScan 750 K Array according to the manufacturer’s instructions (Thermo Fisher Scientific). The obtained raw data were analyzed by Chromosome Analysis Suite 4.2. The segment’s filter was set to 100 kb and 50 markers for CNV and to 1 Mb and 50 markers for loss of heterozygosity. No chromosomal micro-duplication or deletion was detected (including chromosome 11q3.1–11q3.2 region; Figure 1A).

### Whole-Exome Sequencing

Recently, WES had been demonstrated to be helpful for the genetic diagnosis of a fetus with structural anomalies, especially when a diagnosis cannot be made using routine prenatal testing techniques in a fetus with one or more significant anomalies (Lord et al., 2019; Petrovski et al., 2019; Monaghan et al., 2020). To rule out other potential genetic issues, the couple decided to receive a trio WES analysis for the current fetus. Based on the same DNA from chorionic villus tissue, WES was performed according to standard procedures. DNA libraries were prepared following the manufacturer’s recommended protocol (TruSeq, Illumina). Sequencing was performed on an Illumina HiSeq 2000. Raw sequencing reads were aligned to the human genome reference assembly GRCh37 decoy using BWA and processed with sambamba view. Variant calling was processed with Sentieon and further filtered by Freebayes. The filtered variants were annotated with SnpEff and filtered against population frequency [Genome Aggregation Database (gnomAD) and Euler Genomics in-house Han Chinese population database]. The pile-up reads overlapping the deletion site in *PACS1* were produced by Golden Helix Genome Browse. The WES analysis revealed a novel multi-exon deletion (EX12_24del) in *PACS1* (Figure 1B).

### Validation and Pedigree Analysis

**FIGURE 2:**
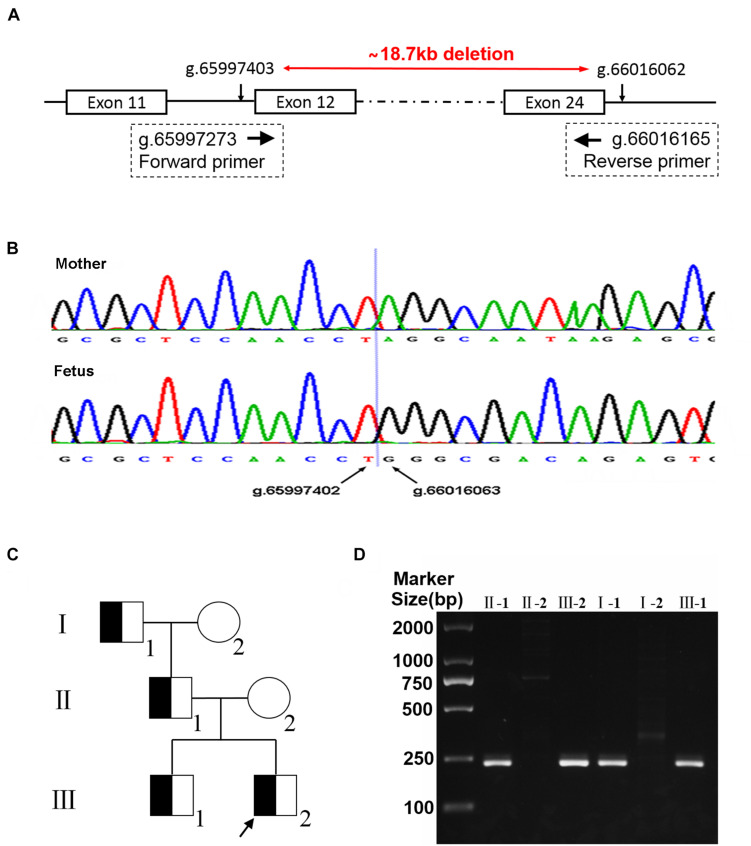
Primers that flank the deletion breakpoints were designed for gap-PCR **(A)**. Deletion of exons 12–24 was validated by Sanger sequencing. An 18,660-bp deletion covering exons 12–24 in *PACS1* was identified (Chr11:65997403-66016062; hg19, GRCh37; **B**). Pedigree of individuals with exons 12–24 deletion in this three-generation family. The variation of the proband (current fetus, III-2) and his elder brother (III-1) was inherited from their father (II-1) and which was derived from their grandfather (I-2; **C**). Electrophoresis of gap-PCR products. The presence of a 233-bp band indicated the carrier of the multi-exon deletion in *PACS1* **(D)**.

This deletion was validated through direct Sanger sequencing. Forward and reverse primers were designed by the site flanking the deletion breakpoints (PF: 5′CAGGCATGATGGCACATGCT3′; PR: 5′TGGGAGTCTGA AATGGAGAAAGG3′; Figure 2A). A deletion with 18,660-bp covering exons 12–24 in *PACS1* was identified (Chr11:65997403-66016062; hg19, GRCh37; Figure 2B). Based on the blood samples collected from the individuals of the family, Sanger sequencing showed that the couple’s first son had the same deletion with the current fetus. As shown in the pedigree (Figure 2C), six individuals were analyzed, and four of them carried the same heterozygous multi-exon deletion encompassing exons 12–24 of *PACS1*. The mutation of the proband was inherited from their father (II-1) and which was derived from their grandfather (I-2; Figure 2C). The products of PCR were electrophoresed, and the presence of a 233-bp band indicated the carrier of this novel multi-exon deletion in *PACS1* (Figure 2D).

## Discussion and Concluding Remarks

*PACS-1* encodes a cytosolic adaptor protein that directs the localization of the trans-Golgi network in transporting certain molecules and proteins (Wan et al., 1998). PACS1 is predominantly a cytoplasmic protein broadly expressed in all organs and highly expressed in brain tissue (Youker et al., 2009). The expression of PACS1 is dynamic during brain development that is up-regulated during the embryonic period and down-regulated after birth (Olson et al., 2018). In 2012, Schuurs-Hoeijmakers and colleagues first identified two unrelated boys with unexplained ID and remarkable facial resemblance possessing an identical *de novo* variation (c.607C > T) in *PACS1* (Schuurs-Hoeijmakers et al., 2012). They also demonstrated that the expression of c.607C > T-mutated *PACS1* mRNA could induce craniofacial defects in zebrafish embryos, which might be through a dominant-negative mechanism (Schuurs-Hoeijmakers et al., 2012). After that, they collected 19 individuals with the same *de novo* c.607C > T variation in *PACS1* from different countries. They also delineated the clinical characteristics of all the patients and suggested that this variation in *PACS1* defines a recognizable syndromic form of DD/ID and dysmorphic facial features (Schuurs-Hoeijmakers et al., 2016). Subsequently, there have been sporadically reported cases with the same *de novo* mutation (c.607C > T) in *PACS1* that caused ID and distinctive facial features (Gadzicki et al., 2015; Stern et al., 2017; Martinez-Monseny et al., 2018; Pefkianaki et al., 2018; Wang et al., 2018; Dutta, 2019; Hoshino et al., 2019; Kurt Colak et al., 2020), except another novel variation (c.608G > A) which affects the same amino acid (Arg203; Miyake et al., 2018). As reviewed and reported recently, more than 50 cases with SHMS have been described in the literature, and all of them have DD/ID and dysmorphic facial features, including 51 postnatal individuals and three prenatal cases (Kurt Colak et al., 2020; Lusk et al., 2020; Seto et al., 2020).

According to the gnomAD, at least 376 non-synonymous variants (347 for missense, 10 for in-frame deletion, nine for in-frame insertion, five for start lost, four for frameshift variant, and one for stop gained) have been registered in the open reading frame of the *PACS1*. However, only two unique missense variants [c.607C > T (p.Arg203Trp) and c.608G > A (p.Arg203Gln)] have been reported as pathogenic in *PACS1* until now. Whether other kinds of variations may result in different *PACS1*-related conditions or even no phenotype remains unknown. To date, five patients with chromosomal duplication (2.26–73.36 Mb) and four patients with chromosomal deletion (1.02–134.18 Mb) covering *PACS1* have been registered in the Database of genomic variation and Phenotype in Humans using Ensemble Resources (DECIPHER). A male patient with a 1.02-Mb deletion (Chr11: 65544038-66563827; hg19, GRCh37) was documented in DECIPHER, with 14 phenotypes involving an abnormal facial shape, delayed speech and language development, and ID were noted (DECIPHER patient: 265913). Even if this is the smallest size of copy number variant (1.02 Mb) that was found in DECIPHER, it still covers at least 54 genes. Although some other cases with copy number variants were also reported with similar clinical features, the exact gene-dosage effect of *PACS1* in these cases was still uncertain (DECIPHER patients: 402716, 398434, and 250851).

As reported previously, the c.607C > T (p.Arg203Trp) substitution locates in exon 4 and affects the furin (cargo)-binding region of the protein which is directly adjacent to the CK2-binding motif (Schuurs-Hoeijmakers et al., 2012). In the present study, the novel heterozygous multi-exon deletion (exons 12–24) mutation in *PACS1* that we reported does not cover the c.607C > T variant but plays the role of coding the C terminal region of the PACS1protein. The proband was noticed and evaluated before his birth for increased NT and the mother’s abnormal pregnancy history. Although chromosomal aneuploidies and CNV examinations were negative, a multi-exon deletion in *PACS1* was detected by WES. Since three carriers of the family were proved to have the same variation with no remarkable physical abnormalities and neurodevelopmental problems, the couple decided to continue the pregnancy after genetic counseling. As reviewed recently by Lusk et al. (2020) and Seto et al. (2020), all the reported patients with SHMS were diagnosed at an early stage of their lives due to the typical clinical characteristics: distinctive facial features, DD, and various degrees of ID. In our report, two of the carriers with multi-exon deletion were adults, and no SHMS-related symptoms and behaviors were noted. So far, only three prenatal cases with c.607C > T in *PACS1* had been described; structural abnormality of different organs such as congenital diaphragmatic hernia, pulmonary hypoplasia, and cardiac anomalies were more likely to be noticed than dysmorphic facial features (Seto et al., 2020). In the present case, the patient received a subsequent routine ultrasound scanning at 24 and 28 weeks of gestation, and no abnormal findings were encountered in the fetus. At the time of this submission, the ages of the proband and his elder brother were 13 and 27 months old, respectively. Both of them showed mild hypermyotonia of the extremities at the age of 3 months, and the symptom was noted to improve as they were 6 months old. However, the proband was found to have left choroid plexus hemorrhage 5 days after birth and subsequently have an increased middle cerebral artery RI and PI at 1 month old. Although his elder brother was reported with no remarkable abnormalities, it is very hard to assert that the hypermyotonia was caused by the deletion mutation in *PACS1*. As far as we can see, none of the reported SHMS cases was recorded with hypermyotonia of the extremities. Approximately 35% of individuals were instead noted with hypotonia, and they were noted to improve over time as well (Seto et al., 2020). At their age of 13 and 27 months old, respectively, the two brothers received a systemic assessment. We listed all the clinical features of our patients in Table 1.

**TABLE 1:** Summary of the clinical features of our patients and the list of reported frequency in patient with SHMS.

Trait	The proband	The proband’s elder brother	Frequency (%) Lusk et al., 2020; Seto et al., 2020; Tenorio-Castano et al., 2021
**General information**			
Age of examination	13 months	27 months	
Gender	Male	Male	
**Growth and feeding**			
Feeding issues	–	–	∼25
Failure to thrive	–	–	14
Microcephaly	–	–	21
Short stature	–	–	12–40
**Neurodevelopmental features**			
intellectual disability	–	–	98–100
Autism spectrum disorder	–	–	21–30
Speech delay	Yes	Yes	76–100
Cognitive impairment	Yes	Yes	38
Temper tant rums/agression	–	–	20
Dysmorphic facial features	Yes	Yes	82–100
bulbous nasal tip	Yes	Yes	21
Short nasal bridge	Yes	Yes	10
Thin upper lip	–	–	18
low-set ears	–	–	15
Arched eyebrows	–	–	15
Wide mouth	–	–	13
Cleft lip	–	–	3∼3.8
**Neurological disorder and behavior**			
Seizures	–	–	50–60
Hypotonia	–	–	∼38
Motor delay	–	–	∼38
Hypermyotonia	Mild at the age of 3 months and recovered over time	Mild at the age of 3 months and recovered over time	Not reported
Structural brain abnormalities	–	–	26–65
**Skeletal abnormalities**			
Pectus excavatum	–	–	8–9.8
Scoliosis	–	–	∼7
Abnormal skull shape	–	–	18–21
**Others**			
Eye abnormalities	–	–	20–33
Congenital heart defect	–	–	13–42
Kidney abnormality	–	–	7–7.7
Cryptorchidism/small testes	–	–	∼28
Choroid plexus hemorrhage	Yes (after birth)	–	Not reported
Transient hyperbilirubinemia	Yes (after birth)	–	Not reported
Increased nuchal translucency	Yes	–	Not reported

**FIGURE 3:**
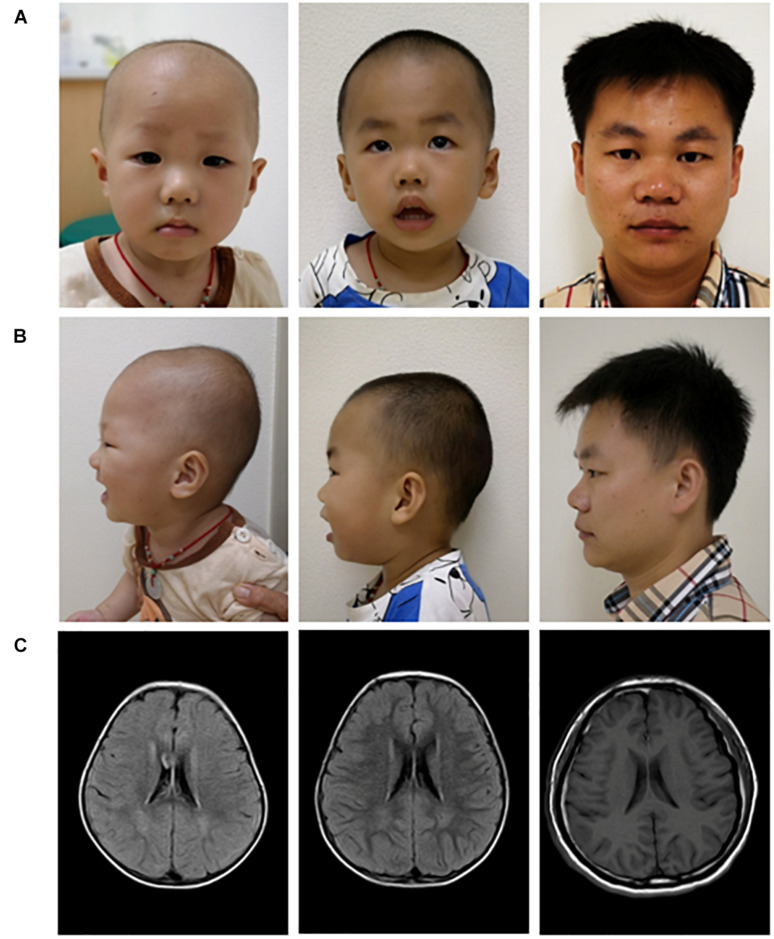
Facial appearance **(A,B)** and brain MRI images **(C)** of three carriers with the multi-exon deletion in *PACS1.* Pictures of the proband (left), the elder brother of the proband (middle), and their father (right).

**FIGURE 4:**
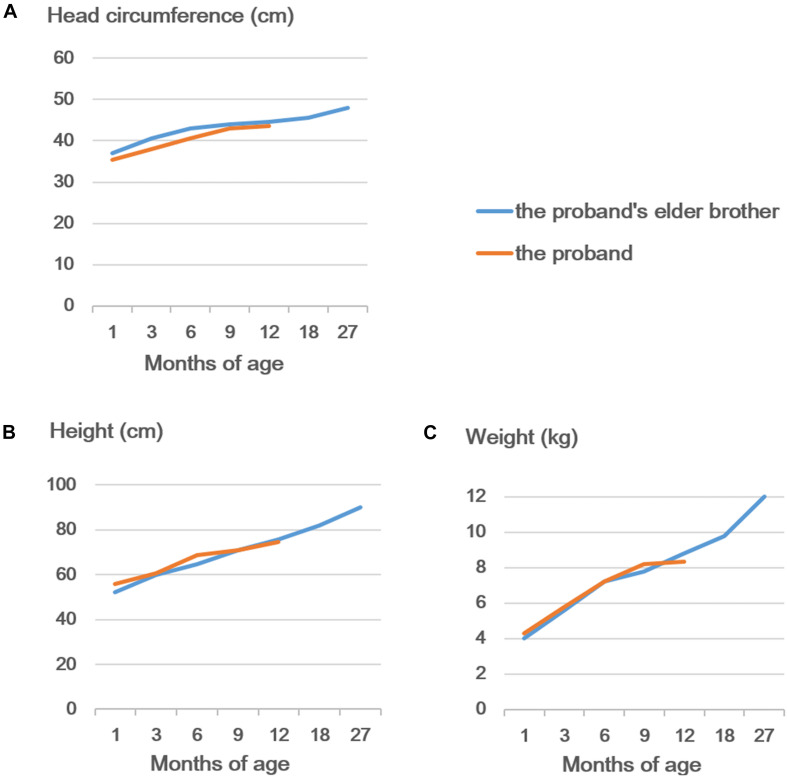
Developmental curves of the head circumference **(A)**, height **(B)**, and weight **(C)** of the proband and his elder brother.

As shown in Figures 3A,B, the brothers had a short nasal bridge and bulbous nose, but not other typical dysmorphic facial features, such as a thin upper lip, low-set ears, full eyebrows, long eyelashes, and a wide mouth. As reported previously, more than 50% of SHMS patients were found to have seizures and structural brain abnormalities (Lusk et al., 2020; Tenorio-Castano et al., 2021). The carriers with multi-exon deletion in *PACS1* were reported with no medical history of seizures, and the brain MRI scan also demonstrated that the brothers and their father had a normal brain structure (Figure 3C). More than 50% of individuals with SHMS have been reported with abnormal height and weight measurements, and 5–10% of these individuals are affected since birth (Lusk et al., 2020; Tenorio-Castano et al., 2021). The data on head circumference and height and weight of the two brothers was collected and analyzed. Both brothers were reported with no feeding problems; according to the growth curves of head circumference (Figure 4A), height (Figure 4B), and weight (Figure 4C), no failure of growth was observed. Biochemical assessments of renal and liver function were also performed; the brothers had normal plasmatic bilirubin and transaminases. Mild speech and cognitive delay were noticed in the brothers. Like the patients with SHMS, our patient also showed that the language skills were more severely affected than the motor skills (Lusk et al., 2020). We think that these two conditions will improve over time as their father and grandfather had normal language and cognitive competence, with no congenital malformations, ID, and neurological abnormalities. Their grandfather was a retired worker, their father graduated from a university with a bachelor’s degree and is now working as an architectural designer in a company. The follow-up observation and assessments of these two boys will be continued.

In the present study, we first identified a novel multi-exon deletion in *PACS1* in four individuals in a three-generation family. Our study might supplement the spectrum of *PACS1*-NDD and hint a possibility that loss-of-function variation in *PACS1* does not contribute to typical SHMS. From our point of view, *PACS1*-NDD could be categorized as a group of diseases in which different variations are associated with different phenotypes. These cases that we show in this study suggest that the effects of loss-of-function mechanism are not as severe as the typical SHMS caused by the dominant-negative or gain-of-function manners. However, more detailed functional experiments are still needed to confirm this hypothesis and establish a genotype–phenotype relationship.

## Data Availability Statement

All datasets generated for this study are included in the article/supplementary material.

## Ethics Statement

The studies involving human participants were reviewed and approved by Institutional Review Board of the Guangdong Women and Children Hospital. Written informed consent to participate in this study was provided by the participants’ legal guardian/next of kin. Written informed consent was obtained from the individual(s), and minor(s)’ legal guardian/next of kin, for the publication of any potentially identifiable images or data included in this article.

## Author Contributions

All authors have materially participated in the study and manuscript preparation. YL collected the data, designed the work, and drafted the manuscript. HD analyzed the clinical data, conducted the follow-up evaluation work, and revised the manuscript. TY collected the data and participated in the follow-up evaluation work. LL designed and performed the validation experiments. LY, FL, and YuZ participated in Sanger sequencing and database search. YH participated in WES data analysis and the interpretation of the data. WH conducted the CMA analysis. YaZ and AY analyzed all the data, conceived the work, and revised the manuscript. All the authors have proved the final article.

## Conflict of Interest

The authors declare that the research was conducted in the absence of any commercial or financial relationships that could be construed as a potential conflict of interest.

## Publisher’s Note

All claims expressed in this article are solely those of the authors and do not necessarily represent those of their affiliated organizations, or those of the publisher, the editors and the reviewers. Any product that may be evaluated in this article, or claim that may be made by its manufacturer, is not guaranteed or endorsed by the publisher.
